# Three-year follow-up of posterior chamber phakic intraocular lens with a central port design after deep anterior lamellar keratoplasty

**DOI:** 10.1186/s40662-022-00306-1

**Published:** 2022-09-07

**Authors:** Belén Alfonso-Bartolozzi, Carlos Lisa, Luis Fernández-Vega-Cueto, Begoña Baamonde, David Madrid-Costa, José F. Alfonso

**Affiliations:** 1 Fernández-Vega Ophthalmological Institute, Avda. Dres. Fernández-Vega 114. 33012, Oviedo, Spain; 2 Clinical and Experimental Eye Research Group (CEER), Optometry and Vision Department, Faculty of Optics and Optometry, Universidad Complutense de Madrid, Madrid, Spain

**Keywords:** Deep anterior lamellar keratoplasty, DALK, Implantable collamer lens, ICL

## Abstract

**Background:**

To evaluate clinical outcomes of the Visian implantable collamer lens (ICL) with a central port to correct myopia and astigmatism after deep anterior lamellar keratoplasty (DALK) for keratoconus throughout 3 years of follow-up.

**Methods:**

This study included 20 eyes of 20 patients that underwent V4c ICL (13 eyes with a spherical ICL and 7 eyes with a toric ICL) implantation after DALK. Uncorrected (UDVA) and corrected (CDVA) distance visual acuities, refraction, intraocular pressure (IOP), endothelial cell density (ECD), and vault were analyzed.

**Results:**

The mean UDVA improved from the preoperative 1.18 ± 0.33 logMAR to 0.25 ± 0.14 logMAR at 6 months after surgery (*P* < 0.0001) and remained unchanged throughout the whole follow-up (*P* = 0.4). All eyes gained lines of CDVA compared to preoperative values. At the last follow-up visit, all eyes achieved CDVA of 0.2 logMAR or better and 13 eyes (65%) 0.1 logMAR or better. At 6 months post-surgery, all eyes (100%) had a spherical equivalent within ± 1.50 D, and 19 (95%) within ± 1.00 D. The mean manifest spherical equivalent was stable over the postoperative follow-up (*P* = 0.25). No significant increase in IOP occurred in any case throughout the 3 years of follow-up. The loss in ECD from the preoperative baseline at the last follow-up visit was 2.27%.

**Conclusions:**

The clinical outcomes suggest that the V4c ICL implantation for correction of myopia and regular astigmatism in post-DALK eyes was satisfactory in terms of effectiveness, safety, and stability during 3 years of follow-up.

## Background

Deep anterior lamellar keratoplasty (DALK) is becoming the preferred option for corneal transplantation in patients affected by a corneal disease in which the endothelium is healthy and can be preserved [1]. However, residual postoperative refractive error may represent a significant limiting factor for visual rehabilitation. The mean spherical equivalent reported after DALK ranges from − 6.54 to − 1.50 D and the mean refractive astigmatism from 2.25 to 4.55 D [2–7]. Hence, the patient’s visual rehabilitation might be limited, even in uncomplicated DALK, due to high residual refractive error and/or anisometropia. Conservative options such as contact lenses or spectacles can be considered for the management of postoperative ametropia. However, anisometropia or high degrees of refractive error corrected with spectacles may not be well tolerated. Furthermore, some patients may experience contact lens intolerance, difficulties with lens handling or lack of motivation for contact lenses fitting. In these patients, several surgical refractive procedures have been studied to improve visual rehabilitation.

The Visian implantable collamer lens (ICL, Staar Surgical AG, Nidau, Switzerland) implantation has been demonstrated to be an effective, safe, and predictable surgical procedure to correct myopia, astigmatism, and hyperopia [8–10]. However, the knowledge is limited to a few studies in patients with a previous penetrating keratoplasty (PKP) or DALK [11–16]. Although these studies have shown promising visual and refractive outcomes, the ICLs evaluated were the former models and the follow-up periods were limited, not spanning more than 24 months. The potential complications associated with ICL implantation increase with time [17–20]. The V4c ICL model (V4c), developed by Shimizu et al. [21], incorporates a central port design that allows the flow of the aqueous fluid through the lens [22, 23], preserving the normal physiology of the anterior segment of the eye and preventing potential complications compared to the previous ICL models [24]. This new design with the central hole could be particularly beneficial in patients with a keratoplasty, minimizing what could represent a potential added risk for cataract development or compromise graft health. This study aims to evaluate clinical outcomes throughout a 3-year follow-up of the ICL V4c implantation in eyes with previous DALK.

## Methods

This retrospective, observational study evaluated eyes with previous DALK and subsequent implantation of the myopic or toric V4c model (STAAR Surgical Inc) to correct the refractive error at the Fernández-Vega Ophthalmological Institute, Oviedo, Spain. This study followed tenets of the Declaration of Helsinki and full ethical approval from the institute was obtained. After receiving a complete description of the possible consequences of surgery, all patients provided informed consent.

The indication for DALK was advanced keratoconus in all cases. All DALK surgeries were performed by the same surgeon (JFA) using Anwar’ technique [25]. The time between the DALK procedure and ICL implantation to correct the residual refractive was at least 6 months after complete suture removal. Before the ICL implantation, all the patients were phakic, had a clear corneal graft, refractive stability for at least 6 months after all sutures were removed, endothelial cell density (ECD) greater than 1500 cells/mm^2^, and a minimum corneal thickness of 400 µm. None of the patients had ocular or systemic diseases with a potential impact on graft survival. Furthermore, patients had met the general criteria for ICL implantation: refractive error in the range correctable with the V4c ICL, anterior chamber depth (ACD) greater than 3.0 mm measured from the corneal endothelium to the anterior lens capsule.

Eyes with a topographic cylinder of more than 3.00 D and regular topographic astigmatism were implanted with a toric ICL. In eyes with a topographic cylinder less than 3.00 D, a spherical ICL was implanted. In these cases, to reduce the topographic cylinder, clear corneal incisions (CCI) were performed on the steep axis. In eyes with a topographic cylinder between 0.75 and 1.25 D, one CCI (3.0–3.2 mm, respectively); in eyes with astigmatism between 1.50 and 2.50 D, two opposite CCI (3.0 mm for 1.50 D and 3.2 mm for astigmatism between 1.75 and 2.50 D) were carried out in the steep axis. All incisions were performed with a bevel-up steel blade (Equipsa S.A., Madrid, Spain). ICL power calculation was performed using a modified vertex formula provided by the manufacturer (Staar Surgical) with a target of emmetropia as postoperative refraction. The size of the ICL was individually calculated for each eye based on the measurements of horizontal white-to-white (WTW) distance, ACD and angle-to-angle distance obtained with anterior segment optical coherence tomography (AS-OCT, Visante, Carl Zeiss Meditec AG).

All surgeries were carried out by the same surgeon (JFA) according to the standard procedure previously described [26, 27]. Postoperative follow-up was 3 years in all cases. The study evaluated the outcomes at preoperative, 6, 12, and 36 months postoperative visits. The examinations included measurement of uncorrected (UDVA) and corrected (CDVA) distance visual acuities, manifest refraction, slit-lamp examination, intraocular pressure (IOP) measured by Goldmann applanation tonometry, ECD (SP 3000P, Topcon Europe) and fundoscopy. The central distance between the ICL and the crystalline lens (vault) was assessed using AS-OCT. The vault between the crystalline lens and the ICL was measured perpendicular to the lens apex or at the narrowest point.

Data analysis was performed using SPSS for Windows, version 14.0 (SPSS Inc., Chicago, IL). Normality was first checked with the Kolmogorov-Smirnov test and a repeated-measures analysis of variance (ANOVA) with a Bonferroni post hoc test was performed thereafter. Differences were considered statistically significant when the *P* value was less than 0.05.

## Results

This study included 20 eyes of 20 patients (15 men and 5 women). The mean time between the DALK procedure and ICL implantation to correct the refractive error was 26.2 ± 12.27 months (range 14–55 months). All patients completed the follow-up period of 3 years and attended all the follow-up visits. Table 1 summarizes preoperative demographic data of the patients and ICL characteristics. Thirteen eyes (65%) were implanted with a spherical ICL and 7 eyes (35%) with a toric ICL. The distribution of the lens sizes implanted were 13.7 mm in 1 eye (5%), 13.2 mm in 10 eyes (50%), 12.6 mm in 5 eyes (25%) and 12.1 mm in 4 eyes (20%).

**Table 1:** Preoperative patient demographics and ICL characteristics

	Mean ± SD	Range (min, max)
Age (years)	39.85 ± 10.40	(25, 62)
Refraction sphere (D)	− 5.78 ± 3.87	(− 15.00, − 0.50)
Refraction cylinder (D)	− 2.36 ± 1.40	(− 5.00, − 0.50)
Spherical equivalent (D)	− 6.96 ± 3.89	(− 16.13, − 2.25)
Minimum keratometry (D)	43.95 ± 2.00	(40.75, 48.00)
Maximum keratometry (D)	46.78 ± 2.19	(42.25, 51.00)
Keratometric cylinder (D)	2.83 ± 1.57	(1.00, 6.00)
Corneal thickness (μm)	507 ± 64	(407, 581)
ACD (mm)	3.49 ± 0.25	(3.00, 3.93)
WTW (mm)	12.26 ± 0.48	(11.45, 13.27)
ATA (mm)	11.94 ± 0.42	(11.00, 13.20)
ECD (cells/mm^2^)	2173 ± 533	(1527, 3347)
IOP (mmHg)	12.65 ± 1.66	(10, 16)
ICL sphere power (D)	− 7.93 ± 3.98	(− 16.50, − 3.50)
ICL toric power (D)*	4.36 ± 0.99	(3.50, 6.00)

*ICL* = implantable collamer lens; *D* = diopters; *ACD* = anterior chamber depth; *WTW* = white-to-white; *ATA* = angle-to-angle; *ECD* = endothelial cell density; *IOP* = intraocular pressure; *SD* = standard deviation. *For 7 eyes that were implanted with a toric ICL

**Fig. 1:**
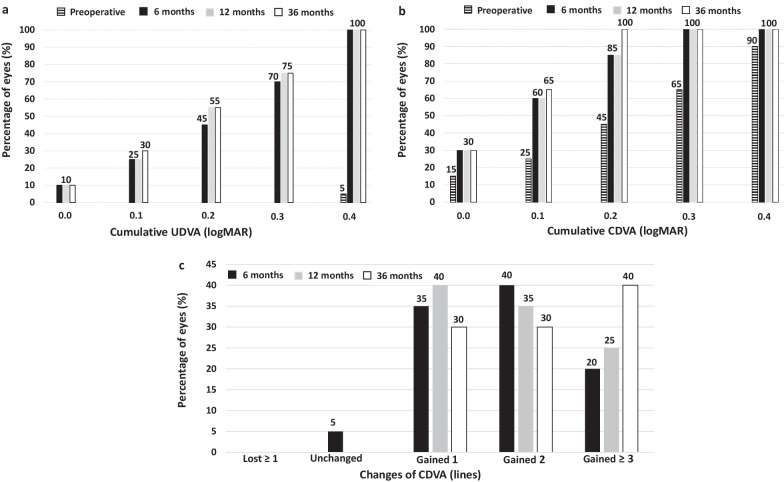
Cumulative uncorrected distance visual acuity (UDVA) (**a**), and corrected distance visual acuity (CDVA) (**b**), at 6, 12 and 36 months post-surgery. **c** Changes in CDVA between preoperative and each postoperative visit

The UDVA improved from 1.18 ± 0.33 logMAR preoperatively to 0.25 ± 0.14 logMAR at 6 months after surgery (*P* < 0.0001) and subsequently remained stable throughout the whole follow-up period (0.25 ± 0.13 logMAR and 0.24 ± 0.13 logMAR, at 12 and 36 months, respectively, *P* = 0.4). The efficacy index (mean postoperative UDVA/mean preoperative CDVA) was 1.06, 1.05, and 1.08 at 6, 12, and 36 months after surgery, respectively. At the last follow-up visit, the UDVA was 0.3 logMAR (20/40) in 15 eyes (75%) (Fig. 1a). The mean CDVA increased from the preoperative 0.28 ± 0.15 logMAR to 0.14 ± 0.09 logMAR at 6 months after surgery (*P* = 0.003) and remained unchanged over the whole follow-up period (0.13 ± 0.10 logMAR and 0.11 ± 0.08 logMAR, at 12 and 36 months, respectively, *P* = 0.09). At the last visit, all eyes achieved CDVA of 0.2 logMAR or better (≥ 20/32) and 13 eyes (65%) had a value of 0.1 logMAR or better (≥ 20/25) (Fig. 1b). Furthermore, all eyes gained lines of CDVA compared to preoperative values (Fig. 1c). The safety index (ratio between the postoperative CDVA at the last visit and the preoperative CDVA) was 1.40.

**Fig. 2:**
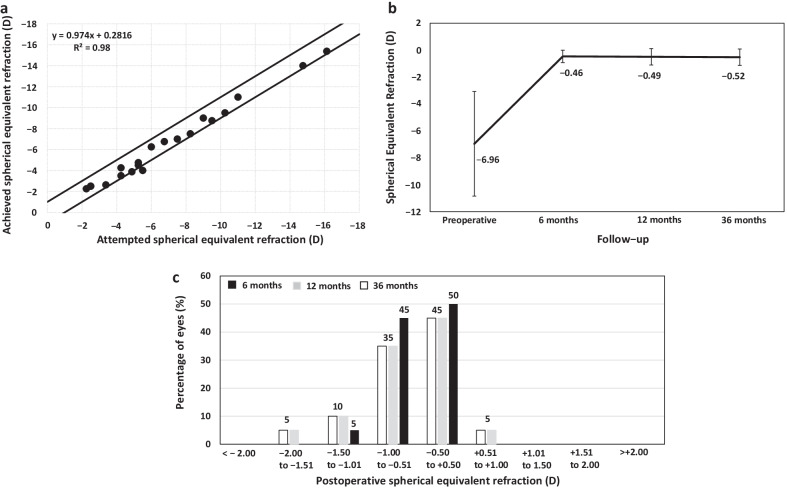
Attempted versus achieved spherical equivalent at 6 months postoperatively (predictability) (**a**), variation of manifest spherical equivalent over the follow-up (stability) (**b**), and accuracy of manifest spherical equivalent at each postoperative follow-up visit

Figure 2a displays the correlation analysis between attempted versus achieved spherical equivalent refraction at 6 months post-surgery. All eyes (100%) had a spherical equivalent within ± 1.50 D and 19 (95%) within ± 1.00 D. The mean manifest spherical equivalent remains stable over the postoperative follow-up (*P* = 0.25) (Fig. 2b). At 12 and 36 months, 17 eyes (85%), had a spherical equivalent within ± 1.00 D (Fig. 2c). In the 7 eyes implanted with toric ICL, the refractive cylinder 6 months after surgery was ≤ 0.50 D. In 6 out of 7 eyes, the refractive cylinder was unchanged over follow-up. In one eye (14.8%), the ICL was rotated between the 6- and 12-month follow-up visits. This ICL rotation induced mixed astigmatism (+ 1.50 − 2.00 × 20°), leading to a decrease of 2 lines of UDVA, but CDVA remained unchanged. The vault in the 12-month visit was 700 μm. As the CDVA was maintained, and the induced mixed was well-tolerated with spectacles, no re-centering maneuverer or ICL exchange for a larger size was performed. The visual and refractive outcomes remained unchanged between the 12- and 36-month follow-up visits.

**Fig. 3:**
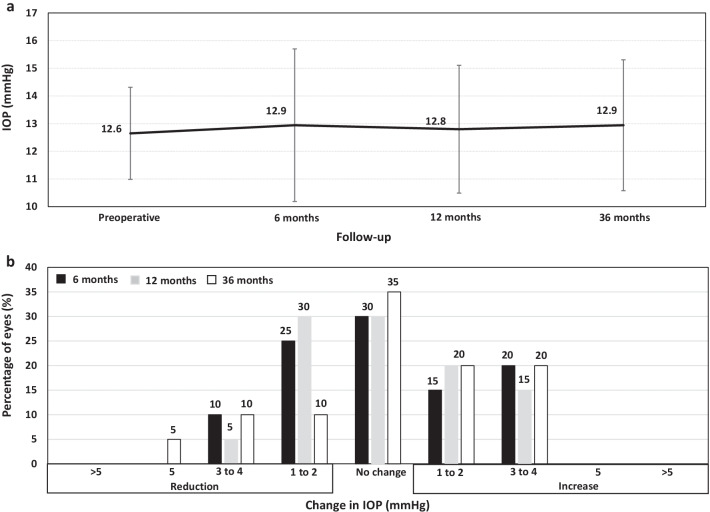
Changes in the mean intraocular pressure (IOP) over the whole follow-up (**a**), and variation in IOP between preoperative and each postoperative follow-up visit (**b**)

The IOP was stable over the whole follow-up (12.6 ± 1.66 mmHg preoperatively and 12.9 ± 1.66 mmHg, 12.8 ± 2.31 mmHg, and 12.9 ± 2.36 mmHg at 6, 12, and 36 months, respectively, *P* = 0.25) (Fig. 3a). At the end of the follow-up, the largest proportion of the eyes experienced a reduction or maintenance of the IOP compared with the preoperative value (12 eyes, 60%), 4 eyes (20%) showed an increased 1–2 mmHg, and 2 eyes (20%) had an increased 3–4 mmHg (Fig. 3b). No significant increase in IOP (> 20 mmHg or an increase higher than 5 mmHg) occurred in any case throughout the 36 months of follow-up.

**Fig. 4:**
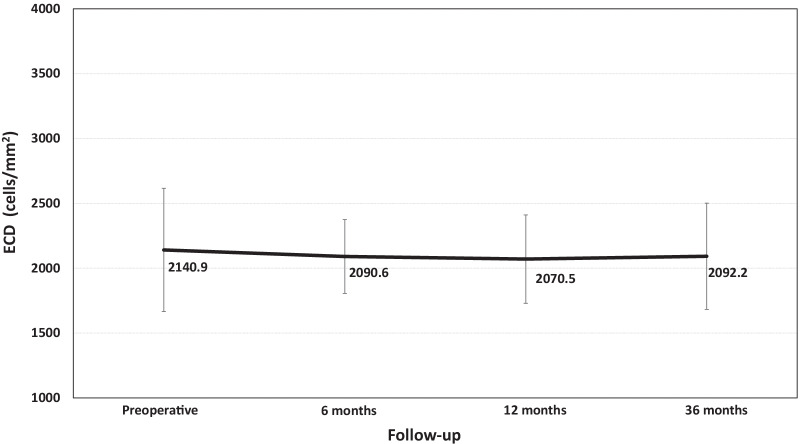
Mean endothelial cell density (ECD) (cells/mm^2^) throughout the follow-up

Regarding the ECD, there were no significant changes at any time of follow-up (*P* = 0.1) (Fig. 4). The loss in ECD from the preoperative compared with the last follow-up visit was 2.27%.

**Fig. 5:**
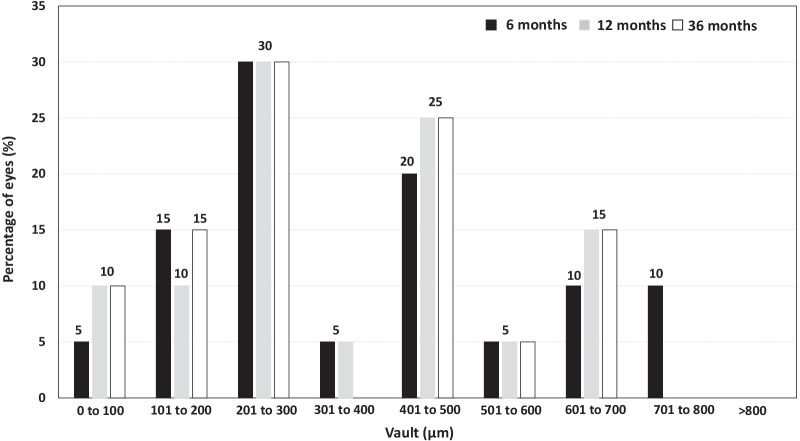
Distribution of eyes according to the vault, measured in microns, at 6, 12, and 36 months postoperatively

The mean postoperative vault between the crystalline lens and ICL under mesopic lighting conditions was slightly reduced among visits (373 ± 217 µm, 361 ± 207 µm, and 345 ± 193 µm at 6, 12, and 36 months postoperatively, respectively). Multiple comparisons showed statistically significant differences among all postoperative visits (*P* = 0.03). The analysis of the postoperative distribution of the vault (Fig. 5) showed that no eyes had a vault higher than 800 µm at any visit, and approximately 20% of eyes had a vault lower than 200 µm throughout the follow-up.

There were no intraoperative complications, and no eyes required ICL explantation or exchange. Throughout the follow-up, no cases of anterior subcapsular opacity, cataract, pigment dispersion glaucoma, pupillary block, or other vision-threatening complications were reported. As previously detailed, one eye implanted with toric ICL had an ICL rotation.

## Discussion

In the cohort analyzed, the post-DALK (before ICL implantation) mean spherical equivalent was − 6.96 ± 3.89 D and the mean refractive cylinder − 2.36 ± 1.40 D. These results agree with those previously reported [2–7] and indicate that whether the refractive error is not well tolerated with spectacles or contact lenses or induces anisometropia may lead to unsatisfactory visual rehabilitation. To overcome this, a variety of refractive surgical procedures have been evaluated. Corneal refractive procedures have shown encouraging results [28–31], however, these options were not appropriate in this group of eyes because of high myopia and astigmatism, dry eye and/or low corneal thickness for the attempted refractive error.

The results of this study of 20 eyes that underwent V4c ICL implantation to correct myopia or astigmatism after DALK were satisfactory. The efficacy index of the procedure was higher than 1.0 at all follow-up visits, which implies that the postoperative UDVA was equal to or better than preoperative CDVA. Furthermore, at the last follow-up visit, all eyes had gained lines of CDVA compared to preoperative values (Fig. 1c). The UDVA was 0.3 logMAR (20/40) in 15 eyes (75%) at the last follow-up visit and all eyes achieved CDVA of 0.2 logMAR or better (≥ 20/32). The safety index was 1.40. It is important to note that after DALK, the cornea may present optical irregularities such as irregular astigmatism or increased higher-order aberrations, which might represent a potential factor in limiting the visual restoration after ICL implantation. In those cases, a combined procedure, such as intrastromal corneal ring segments implantation for corneal regularization and subsequently ICL for the residual refractive error correction, might be a better approach. Our group found this approach very effective in keratoconus patients [32], however, it should be analyzed in post-DALK eyes to evaluate the effectiveness and safety in these cases.

The visual outcomes were stable throughout the follow-up, confirming the efficacy, safety, and stability of the procedure. On the contrary to what we found in this study in post-DALK eyes, the efficacy index of the V4c ICL implantation in virgin corneas may worsen over time owing to an axial length elongation and consequently increase in myopia and decrease in UDVA [27]. It is noteworthy that this procedure is carried out at an older age in post-DALK patients, and thus, the risk of axial length elongation is lower. However, in younger patients, this aspect should be assessed preoperatively. Although LASIK and PRK have shown efficacy and safety in post-DALK [28–31], and a laser touch-up can be effectively and safely planned, this risk should be evaluated and explained to the patients.

Regarding refractive outcomes, at 6 months, the mean spherical equivalent was − 0.46 ± 0.46 D, with 95% of the eyes with a spherical equivalent within ± 1.00 D. In the 7 eyes implanted with toric ICL, the refractive cylinder 6 months after surgery was ≤ 0.50 D. Therefore, these results show that spherical and toric V4c ICL are capable of correcting for myopia and astigmatism effectively in post-DALK eyes. Furthermore, the refractive outcomes remained stable over the 3 years of follow-up in all cases except in one eye implanted with toric ICL. In this eye, the refraction changed from plano at 6 months to + 1.50 − 2.00 × 20° at 12 months, and the UDVA dropped 2 lines, while CDVA remained unchanged. After slit-lamp examination, a rotation of 15 degrees from the original position was observed. As the CDVA was maintained, and the induced mixed astigmatism was well-tolerated with spectacles, we opted for a conservative strategy protecting the graft from another surgical procedure and no re-centering maneuverer or ICL exchange for a larger size was performed. It should be noted that our criterion for toric ICL implantation was regular astigmatism confirmed by topography. The combination of toric ICL rotated off-axis with an irregular cornea could cause a decrease not only in UDVA but also in CDVA thereby requiring another surgical intervention for ICL re-centering or ICL exchange. It should be kept in mind that unlike patients with a virgin cornea whose aim must be to achieve emmetropia, the main objective for post-DALK patients on the other hand, should be to reduce the refractive error as much as possible to avoid anisometropia and improve the spectacle tolerance. Considering this, our study showed that the implantation of spherical ICLs combined with CCI is also an excellent alternative to reduce myopia and astigmatism, avoiding the possible complications derived from the toric rotation of the ICL.

No direct comparison with previous studies of this model of ICL in post-DALK eyes is possible because, to the best of our knowledge, this is the first study to evaluate V4c ICL in eyes with a previous DALK. Four previous studies have evaluated the correction of residual refractive error with a former model of ICL in post-PKP eyes and only two studies in post-DALK eyes. Akcay et al. [11] reported that one eye underwent toric ICL implantation to treat high myopia and astigmatism after PKP. Alfonso et al. [12] evaluated 15 eyes that had spheric or toric ICL (9 eyes with a spherical ICL and 6 eyes with a toric ICL) after PKP with a follow-up of 2 years. Mehta et al. [13] studied three post-PKP with a follow-up ranging between 3 and 14 months. Iovieno et al. [14], in turn, reported the outcomes of toric ICL implantation in 7 post-keratoplasty eyes (6 PKP and 1 DALK) over a follow-up range between 4 and 22 months. Qin et al. [15] investigated the implantation of toric ICL in 9 DALK cases over 2 years of follow-up. In the Alfonso et al.’s study [16], the ICL was implanted as Piggyback IOL for the correction of residual refractive error in 7 post-DALK pseudophakic eyes, with a mean follow-up of 13.32 ± 13.57 months. All these studies concluded that the ICL implantation significantly reduced the refraction with a high accuracy rate (with most eyes within ± 1.00 D of the desired refraction), and a significant improvement in UDVA and CDVA and most of the eyes maintaining or improving the preoperative CDVA. Not surprisingly, our visual and refractive outcomes with the V4c ICL are in line with those previously published with the prior ICL model, but our study also confirms that these results remained stable over a longer follow-up period than those previously reported. Hence, these values confirm the excellent visual and refractive results of this procedure in post-keratoplasty eyes.

However, we should be cautious and consider that any intraocular procedure may have associated potential adverse events. The rate of adverse events with the previous ICL models (such as cataracts, ECD loss, pigment dispersion syndrome) increased with time [17–20, 33] and the most extended follow-up in post-keratoplasty eyes reported up to now not spanning more than 2 years. The incidence of complications has significantly decreased with the V4c model in comparison to those reported with the previous ICL models (non-hole ICL). The central hole of the V4c ICL offers surgical advantages over previous ICL models (non-hole ICL) models, reducing ocular trauma and simplifying the surgery since no preoperative iridotomy or intraoperative iridectomy is necessary to prevent IOP increase [33, 34]. Furthermore, the hole-equipped ICL allows for maintaining the normal physiology of the anterior segment of the eye since aqueous humor may flow through the hole of the lens, preventing potential complications [24].

Fernandes et al. [33] reported that the prevalence of cataracts with the previous models of ICL was 5.2%, while this percentage dropped to almost 0% (0.17%) with V4c ICL [22]. The main risk factor for ICL-induced cataracts with non-hole ICL was a vault lower than 200 µm [33]. In our study in post-DALK eyes, we did not find cataracts, although approximately 20% of the cases had a vault lower than 200 µm. Recently, our research group reported the most extended follow-up with V4c ICL published up to now in patients with the virgin cornea (84 eyes followed for 7 years) [27], in which approximately 20% of the eyes had a vault lower than 200 µm, and we did not find cataract formation in any cases throughout the follow-up. Hence, it is plausible to suggest that the central hole of the V4c ICL model prevents cataract development, even in eyes with a low vault, confirming the safety of this model [35].

Regarding IOP and ECD, our results did not yield a statistically significant change over the follow-up. The IOP was stable over the 36 months of follow-up. At the end of the follow-up, most eyes showed a reduction or maintenance in IOP from the preoperative value, with no eyes experiencing a significant increase in IOP (> 20 mmHg or an increase higher than 5 mmHg) throughout the whole follow-up. In turn, the loss in ECD from the preoperative at the last follow-up visit was 2.27%. This finding agrees with those previously reported, suggesting that the V4c ICL does not promote significant ECD loss over time [24]. In addition to the benefits of the central hole design to prevent an IOP increase and ECD loss, it should note that no eyes showed a vault higher than 800 µm at any follow-up visit, which might represent a potential risk factor [36].

All these findings suggest that the central hole design of the V4c ICL might provide safety to the outstanding visual and refractive outcomes previously reported with the previous model, and it is essential in post-keratoplasty eyes. However, despite these encouraging results, it is important to note the limitations of our study; its retrospective design and the lack of a control group. Therefore, further long-term studies with a randomized comparative prospective design are necessary to evaluate the possible complications of this technique in post-DALK eyes.

## Conclusion

Our clinical outcomes suggest that the V4c ICL implantation for correction of myopia and regular astigmatism in post-DALK eyes was satisfactory in terms of effectiveness, safety, and stability during 3years of follow-up, which finally indicates its viability as a surgical option for the residual refractive error correction after DALK.

## Data Availability

Not applicable.

## Abbreviations

DALK: Deep anterior lamellar keratoplasty
PKP: Penetrating keratoplasty
ICL: Implantable collamer lens
ECD: Endothelial cell density
ACD: Anterior chamber depth
CCI: Clear corneal incisions
WTW: White-to-white
AS-OCT: Anterior segment optical coherence tomography
UDVA: Uncorrected distance visual acuity
CDVA: Corrected distance visual acuity
IOP: Intraocular pressure
